# Supplementary material for: Identification of Freezing-Responsive microRNAs and Their Targets in Chinese Jujube by Small RNA and Degradome Sequencing
Source: Int J Mol Sci. 2026 Sep 4;27(17):7912. doi: 10.3390/ijms27177912 (PMC13566890; doi:10.3390/ijms27177912)
Supplement: Supplementary file 1 [file ijms-27-07912-s001.zip › Supplementary Material S2/novel_miR_76.pdf]

Provisional ID	:	novel_miR_76
Score total	:	660.3
Score for star read(s)	:	3.9
Score for read counts	:	652.7
Score for mfe	:	2.1
Score for randfold	:	1.6
Score for cons. seed	:	
Total read count	:	1292
Mature read count	:	1185
Loop read count	:	75
Star read count	:	32

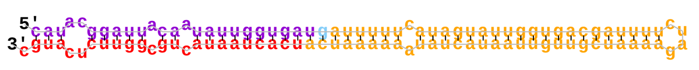

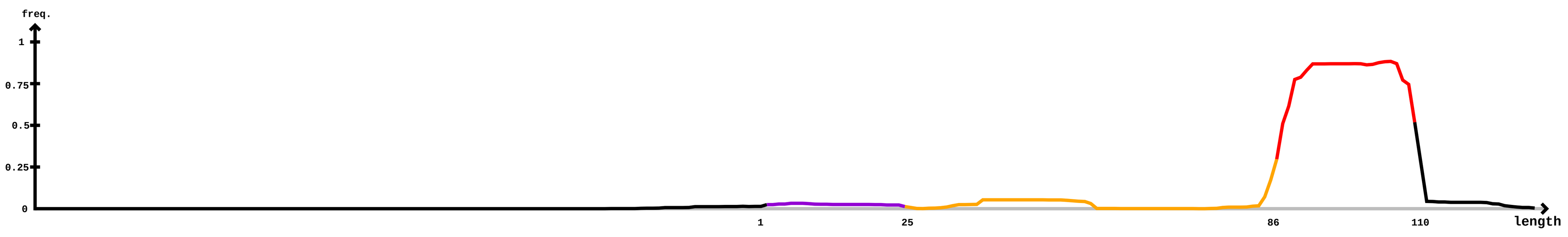

Star Mature

```
5'- aaauaauaagcucauaucuuguguucaaaguuugaaacuaaaaccccaauuucuuaauuuaucaauauauauauauauauguauauauguauaccguucgucuaugguauggacgguuuucauacggauuacaauauuggugaugauuuuucauaguauuggugacgauuuucuagaaaaucguuguuaaauacuauaaaaaaaucaucacuaauacugcgguucucaugcagaccguccucaccauagaau -3' obs
    aaauaauaagcucauaucuuguguucaaaguuugaaacuaaaaccccaauuucuuaauuuaucaauauauauauauauauguauauauguauaccguucgucuaugguauggacgguuuucauacggauuacaauauuggugaugauuuuucauaguauuggugacgauuuucuagaaaaucguuguuaaauacuauaaaaaaaucaucacuaauacugcgguucucaugcagaccguccucaccauagaau exp
```

read	reads	mm	sample
cguucgucuaugguaugga	1	0	LT-3
guUuaugguauggacggu	2	1	LT-3
ucuaugAuauggacgguuuu	1	1	JCK-1
uaugguauggacgguCuuc	1	1	JCK-2
augguauggacgguuuucauG	4	1	JCK-3
uauggacgguuCucauacgga	1	1	LT-3
auggacgguuCucauacgg	1	1	LT-3
auggacgguuCucauacgg	2	1	LT-1
auggacgguuuucauGcgga	1	1	JCK-2
auggacgguuuucauaUgga	2	1	LT-2
auggacgguuuucauaUggau	1	1	LT-3
cgguuuucauacggauCa	2	1	LT-1
uuucauacggauCacaauauugg	1	1	JCK-3
uuucauacggauCacaauauugg	1	1	JCK-2
uucauacggauNacaauauugg	1	1	JCK-1
cauacggauuacaauauu	1	0	JCK-2
cauacggauuacaauauuggugau	2	0	JCK-1
cauacggaCuacaauauuggugau	1	1	LT-2
cauacggaCuacaauauuggugau	3	1	JCK-1
cauacggauuacaauauuggugaC	1	1	JCK-2
cauacggauuacaauauuggugaC	2	1	JCK-3
cauacggaCuacaauauuggugau	2	1	JCK-3
cauacggaCuacaauauuggugau	4	1	JCK-2
auacggauuacaGuauuggugа	1	1	LT-3
uacggauuacaGuauuggugа	1	1	LT-1
uacggauuaUaauauuggugа	1	1	LT-1
uacggauuacaauauuggugaC	1	1	JCK-2
uacggauuacaauauuggugaC	1	1	JCK-3
uacggauuacaauauuggugaugG	1	1	JCK-3
cggauuacaGuauuggugа	4	1	JT-2
cggauuacaGuauuggugа	6	1	JT-3
gauuuuucauaguaCuggugacg	1	1	LT-1
uuuucauaguaCuggugacgauuu	3	1	LCK-3

Star Mature

aaauaauaagcucauaucuuguguucaaaguuugaaacuaaaaccccaauuucuuaauuuaucaauauauauauauauauguauauauguauaccguucgucuaugguauggacgguuuucauacggauuacaauauuggugaugauuuuucauaguauuggugacgauuuucuagaaaaucguuguuaauacuauaaaaaaaucaucacuaaauacugcgguucucaugcagaccguccucaccauagaau

uuucauaguauuggugacgauuuuc	1	0	LT-1
uucauaguauuggugacgauu	1	0	JCK-3
uucauaguauuggugacgauu	1	0	JCK-2
uucauaguauuggugacgauuuucu	1	0	LT-1
ucauaguaCuggugacgauuuucu	1	1	JCK-2
ucauaguaCuggugacgauuuucu	1	1	JCK-3
ucauaguauuggugacgauuuucu	2	0	LT-2
ucauaCuauuggugacgauuuucu	2	1	JCK-1
cauaguauuggugacgaAuuu	2	1	LT-2
cauaguauuggugacgauuuu	1	0	LT-2
cauaguaAuggugacgauuuuc	1	1	LT-3
cauaguauuggugaAgauuuucu	4	1	LCK-3
cauaguauuggugacgauuuucu	1	0	JCK-2
cauaguauuggugacgauuuucu	1	0	JCK-1
cauaguauuggugacgauuuucu	1	0	JCK-3
auaguauuggugacgauu	1	0	LT-1
auaguaCuggugacgauuu	1	1	JCK-3
auaguauuggugacgauuuu	1	0	JCK-3
auaguauuggugacgauuuuc	1	0	JCK-1
auaguauuggugacgauuuucu	1	0	LT-1
auaguauuggugacgauuuucu	1	0	JCK-2
auaguauuggugacgauuuucuG	3	1	LT-2
aguGuuggugacgauuuucu	1	1	LT-1
guauuggugacgauuuucuagaaa	1	0	JCK-1
uaAuggugacgauuuucua	21	1	LCK-2
uaAuggugacgauuuucua	17	1	LCK-1
uagaaaaucguCguuaaua	1	1	LT-3
auaaaaaaucaucacuaauacugc	1	0	LT-1
auaaaaaCucaucacuaauacugc	1	1	JCK-2
Aaaaaaaucaucacuaauacugcg	1	1	JCK-3
Gaaaaaucaucacuaauacugcgg	1	1	JCK-3
Gaaaaaucaucacuaauacugcgg	4	1	JCK-1
Gaaaaaucaucacuaauacugcgg	2	1	JCK-2
aaaaaucaucacuaauacugcgU	1	1	JCK-1
aaaaaucaucacuaauacugcgU	2	1	JCK-2
aaucaucacuaauacugcgguCc	1	1	LT-1
aucaucacuaauacugcgguCcuc	1	1	JCK-2
aucaucacuaauacugcgguucuc	5	0	JCK-2
aucaucacuaauacugcgguCcuc	1	1	JCK-3
uUaucacuaauacugcgguucuc	1	1	JCK-1
uUaucacuaauacugcgguucuc	2	1	JCK-2
caucacuaauacugcgguu	1	0	LT-3
caucacuaCuacugcgguu	1	1	LT-2
caucacuaCuacugcgguucu	1	1	LT-3
caucacuaauacugcgguucu	2	0	JCK-1
caucacuaauacugcgguucuc	1	0	LT-1
caucaUuaauacugcgguucuca	2	1	JCK-2
caucacuaauacugcgguucuca	9	0	LCK-3
cauNacuaauacugcgguucuca	1	1	LCK-1
caucacuaauacugcgguucuca	21	0	LCK-1
caucacuaauacugcgguucuca	1	0	LT-2
caucacuaauacugcgguucuca	23	0	LCK-2
caucaUuaauacugcgguucuca	1	1	JCK-1
caucacuaauacugcgguucucau	4	0	JCK-2
caucacuaauacugcgguucucau	1	0	JCK-3
caucacuaauacugcgguucucau	2	0	LT-3
caucaUuaauacugcgguucucau	1	1	JCK-2
caucaUuaauacugcgguucucau	3	1	JCK-3
aucaUuaauacugcgguucuc	1	1	JCK-2
aucaUuaauacugcgguucuc	1	1	JCK-1
aucacuaCuacugcgguucuca	2	1	LT-2
aucacuaauacugcgguucuca	2	0	LT-2
aucaUuaauacugcgguucucau	1	1	JCK-1
aucacuaauacugcgguucucau	1	0	JCK-2
aucacuaauacugcgguucucau	1	0	LT-2
aucacuaauacugcgguucucau	4	0	LT-3
aucaUuaauacugcgguucucau	1	1	JCK-2
aucacuaauacugcgguucucau	1	0	JCK-1
aucacuaCuacugcgguucucaug	2	1	JCK-3
aucacuaauacugUgguucucaug	4	1	JCK-1

```
                                                                                                                                                 Star                                                                                         Mature
aaauaauaagcucauaucuuguguucaaaguuugaaacuaaaaccccaauuucuuaauuuaucaauauauauauauauauguauauauguauaccguucgucuaugguauggacgguuuucauacggauuacaauauuggugaugauuuuucauaguauuggugacgauuuucuagaaaaucguuguuaauacuauaaaaaaaucaucacuaauacugcgguucucaugcagaccguccucaccauagaau
.............................................................................................................................................................................................................aucacuaauacugcgAuucucaug......................   2   1   JCK-3
.............................................................................................................................................................................................................aucacuaauacugcUguucucaug......................   5   1   JCK-2
.............................................................................................................................................................................................................aucacuaCuacugcgguucucaug......................   1   1   JCK-1
.............................................................................................................................................................................................................aucacuaauacugcgguucucaug......................   11  0   JCK-1
.............................................................................................................................................................................................................Cucacuaauacugcgguucucaug......................   1   1   JCK-3
.............................................................................................................................................................................................................aucacuaCuacugcgguucucaug......................   1   1   JCK-2
.............................................................................................................................................................................................................aucacuaauacugcgguuGucaug......................   2   1   LT-1
.............................................................................................................................................................................................................aucacuaauaUugcgguucucaug......................   1   1   JCK-3
.............................................................................................................................................................................................................aucacuaauacugcgguucucaug......................   30  0   JCK-2
.............................................................................................................................................................................................................aucacuaauacuAcgguucucaug......................   2   1   LT-1
.............................................................................................................................................................................................................aucacuaauacugUgguucucaug......................   1   1   LT-1
.............................................................................................................................................................................................................aucacuaauacugcgAuucucaug......................   4   1   JCK-2
.............................................................................................................................................................................................................aucacuaauacugUgguucucaug......................   2   1   JCK-3
.............................................................................................................................................................................................................aucacuaauacugUgguucucaug......................   2   1   JCK-2
.............................................................................................................................................................................................................aucacuaauaUugcgguucucaug......................   1   1   JCK-2
.............................................................................................................................................................................................................aucacuaCuacugcgguucucaug......................   4   1   LT-1
.............................................................................................................................................................................................................aucacuaauacugcgguucucaug......................   5   0   LT-1
.............................................................................................................................................................................................................aucaUuaauacugcgguucucaug......................   1   1   LT-1
.............................................................................................................................................................................................................aucacuaauacugcgguucucaug......................   8   0   LCK-3
.............................................................................................................................................................................................................aucacuaauacugcgguucucaug......................   3   0   LT-2
.............................................................................................................................................................................................................aucacuaauacugcgAuucucaug......................   6   1   JCK-1
.............................................................................................................................................................................................................aucacuaauacugcgguucucaug......................   23  0   JCK-3
.............................................................................................................................................................................................................aucacuaauacugcgguucucaugc.....................   1   0   JCK-2
.............................................................................................................................................................................................................aucacuaauacugcgguucucaugc.....................   2   0   JCK-1
.............................................................................................................................................................................................................aucacuaauacugcgguucucaugc.....................   1   0   JCK-3
..............................................................................................................................................................................................................ucacuaauacugcgguucucG........................   1   1   JCK-2
..............................................................................................................................................................................................................ucacuaauacugcgguucuca........................   1   0   JCK-3
..............................................................................................................................................................................................................ucacuaauacugcgguucuca........................   2   0   JCK-2
..............................................................................................................................................................................................................ucacuaauacugcUguucucaug......................   2   1   JCK-1
..............................................................................................................................................................................................................ucacuaauacugcgguucucaug......................   4   0   JCK-2
..............................................................................................................................................................................................................ucacuaauacugcgguucuUaug......................   1   1   JCK-2
..............................................................................................................................................................................................................ucacuaauacugcgguucucaug......................   2   0   JCK-1
..............................................................................................................................................................................................................ucacuaauacugcgguucuUaug......................   1   1   JCK-1
..............................................................................................................................................................................................................ucacuaauacugcgguucucaug......................   3   0   LT-2
..............................................................................................................................................................................................................ucaUuaauacugcgguucucaug......................   3   1   JCK-1
..............................................................................................................................................................................................................ucacuaauacugUgguucucaug......................   7   1   LCK-3
..............................................................................................................................................................................................................ucacuaauacugcgguucucaug......................   1   0   JCK-3
..............................................................................................................................................................................................................ucacuaauacugcgguucucaugc.....................   42  0   JCK-3
..............................................................................................................................................................................................................ucacuaauacugcgguucucaugc.....................   37  0   JCK-2
..............................................................................................................................................................................................................ucacuaauacugUgguucucaugc.....................   2   1   LT-1
..............................................................................................................................................................................................................ucacuaauacugUgguucucaugc.....................   2   1   LT-2
..............................................................................................................................................................................................................ucacuaauacugcgguucucGugc.....................   1   1   LT-1
..............................................................................................................................................................................................................ucacuaauacugcgguucucaugc.....................   7   0   LT-1
..............................................................................................................................................................................................................ucacuCauacugcgguucucaugc.....................   2   1   LT-1
..............................................................................................................................................................................................................ucaUuaauacugcgguucucaugc.....................   1   1   JCK-1
..............................................................................................................................................................................................................ucacuaauacugcgguucucaugc.....................   16  0   LT-3
..............................................................................................................................................................................................................ucacuaCuacugcgguucucaugc.....................   1   1   LT-2
..............................................................................................................................................................................................................ucacuCauacugcgguucucaugc.....................   1   1   LT-3
..............................................................................................................................................................................................................ucacuaauacugcCguucucaugc.....................   1   1   LT-2
..............................................................................................................................................................................................................ucacuaCuacugcgguucucaugc.....................   1   1   LT-1
..............................................................................................................................................................................................................ucacuaauacugcgguucucaugc.....................   1   0   LT-2
..............................................................................................................................................................................................................ucacuaauacugcCguucucaugc.....................   1   1   LT-3
..............................................................................................................................................................................................................ucacuaauacugcgguucucaugc.....................   22  0   JCK-1
..............................................................................................................................................................................................................ucacuaauacugcggGucucaugc.....................   2   1   LCK-3
..............................................................................................................................................................................................................ucacuCauacugcgguucucaugc.....................   5   1   LT-2
...............................................................................................................................................................................................................cacuaauacugcgguucu..........................   6   0   LCK-3
...............................................................................................................................................................................................................cacuaauacugcgguucuc.........................   1   0   JCK-2
...............................................................................................................................................................................................................cacuaauacugcgguucuc.........................   1   0   JCK-3
...............................................................................................................................................................................................................cacuaauaUugcgguucuc.........................   2   1   LT-3
...............................................................................................................................................................................................................cacuaauacugcggNucuca........................   1   1   LCK-1
...............................................................................................................................................................................................................cacuaauacugcgguucuca........................   1   0   JCK-3
...............................................................................................................................................................................................................cacuaauacugcgguucuca........................   4   0   LCK-3
...............................................................................................................................................................................................................cacuaauacugcgguucuca........................   33  0   LCK-1
...............................................................................................................................................................................................................cacuaauacugcgguucuca........................   29  0   LCK-2
...............................................................................................................................................................................................................cacuaauacugcgguucucau.......................   2   0   JCK-2
...............................................................................................................................................................................................................cacuaauacugcgguucucau.......................   2   0   JCK-3
...............................................................................................................................................................................................................cacuaauacugcgguucucau.......................   1   0   LT-3
...............................................................................................................................................................................................................cacuaauacugcgguucucau.......................   3   0   LT-2
...............................................................................................................................................................................................................cacuCauacugcgguucucaug......................   1   1   JCK-3
...............................................................................................................................................................................................................cacuaauacugUgguucucaug......................   2   1   LT-2
```

Star **Mature**

aaauaauaagcucauaucuuguguucaaaguuugaaacuaaaaccccaauuucuuaauuuaucaauauauauauauauauguauauauguauaccguucgucuaugguauggacgguuuucauacggauuacaauauuggugaugauuuuucauaguauuggugacgauuuucuagaaaaucguuguuaauacuauaaaaaaucaucacuaauacugcgguucucaugcagaccguccucaccauagaau

cacuaauacugcgguucucaug	8	0	JCK-2
cacuaauacugcgguucucauU	2	1	JCK-2
cacuaauacugcgguucucauU	2	1	JCK-3
cacuaauacugcggGucucaug	2	1	JCK-2
cacuaauacugcgguucucaug	1	0	LT-1
cacuaauacugcgguucucaug	6	0	LCK-3
cacuaauacugUgguucucaug	2	1	LT-1
cacuaauacugcgguucucaug	9	0	JCK-3
cacuaauacugcgguucucaug	7	0	JCK-1
cacuaauacugcgguucucauU	1	1	LT-1
cacuaauacugUgguucucaug	1	1	JCK-1
cacuaauacugUgguucucaug	1	1	JCK-3
cacuaauacugcgguucucaug	4	0	LT-2
cacuaauacugcgguucucaugc	25	0	JCK-2
cacuaCuacugcgguucucaugc	2	1	LT-1
cacuaauacugcgguucCcaugc	1	1	LT-1
cacuaauacugcgguucuUaugc	1	1	JCK-3
cacuaauacugcUguucucaugc	2	1	JCK-3
cacuaauacugcCguucucaugc	1	1	LT-3
cacuaauacugcCguucucaugc	1	1	JCK-2
cacuaauacugcgguucucaugc	11	0	LT-2
cacuaauacugcgguucucGugc	1	1	LT-3
cacuaauacugcUguucucaugc	2	1	JCK-1
cacuaauacugcgguucucaugc	15	0	LT-3
cGcuaauacugcgguucucaugc	1	1	LCK-3
cacuaauacugcgguucucaugc	16	0	LT-1
cacuCauacugcgguucucaugc	2	1	JCK-1
cacuCauacugcgguucucaugc	1	1	JCK-2
caUuaauacugcgguucucaugc	2	1	JT-3
cacuaauacugcgguucucaugc	21	0	JCK-3
caUuaauacugcgguucucaugc	3	1	JCK-3
cacuaCuacugcgguucucaugc	6	1	LT-2
caUuaauacugcgguucucaugc	3	1	JT-2
cacuCauacugcgguucucaugc	2	1	LT-1
cacuaauacugcgguucuUaugc	4	1	JCK-1
cacuaauacugcgguucucaugc	17	0	JCK-1
cacuaauacugcgguucucaugc	21	0	LCK-3
caUuaauacugcgguucucaugc	1	1	JCK-1
acuaauacugcgguucuc	2	0	JCK-3
acuaauacugcgguucuc	1	0	JCK-1
acuaauacugcgguucuUa	1	1	LT-3
acuCauacugcgguucuca	2	1	LT-3
aUuaauacugcgguucucau	1	1	JCK-3
acuaauacugUgguucucau	1	1	LT-1
acuaauacugcgguucucGu	1	1	LT-1
acuaauacugcgguucucau	1	0	LT-1
aUuaauacugcgguucucau	1	1	JCK-2
acuCauacugcgguucucaug	3	1	JCK-1
aUuaauacugcgguucucaug	1	1	JCK-3
aUuaauacugcgguucucaug	2	1	JCK-1
acuaauacugUgguucucaug	4	1	JCK-3
acuaauacugcUguucucaug	1	1	LT-1
acuaauacugGgguucucaug	1	1	JCK-3
acuCauacugcgguucucaug	2	1	JCK-3
acuCauacugcgguucucaug	3	1	JCK-2
acuaauacugcgguucucaug	2	0	LT-2
acuaauacugcgguucucauU	1	1	LT-1
acuaauacugcgguucucaug	5	0	JCK-1
acuCauacugcgguucucaug	1	1	LT-1
acuaauacugUgguucucaug	7	1	JCK-2
acuaauacugcgguucucGug	2	1	LT-1
acuaauacugcgguucucaug	30	0	JCK-2
acuaauacugcgguucucaug	14	0	JCK-3
acuaauacugGgguucucaug	3	1	JCK-2
acuaauacugcUguucucaug	3	1	JCK-3
acuaauacugcggGucucaug	2	1	JT-1
acuaauacugcUguucucaug	2	1	JCK-2
acuaCuacugcgguucucaug	1	1	LT-3
aUuaauacugcgguucucaug	1	1	JCK-2
Gcuaauacugcgguucucaugc	1	1	JCK-1

Star Mature

```
aaauaauaagcucauaucuuguguucaaaguuugaaacuaaaaccccaauuucuuaauuuaucaauauauauauauauauguauauauguauaccguucgucuaugguauggacgguuuucauacggauuacaauauuggugaugauuuuucauaguauuggugacgauuuucuagaaaaucguuguuaaauacuauaaaaaaaucaucacuaauacugcgguucucaugcagaccguccucaccauagaau
.........................................................................................................................................................................................................acuaaCacugcgguucucaugc.....................   2    1    LCK-3
.........................................................................................................................................................................................................aUuaauacugcgguucucaugc.....................   2    1    LT-2
.........................................................................................................................................................................................................aUuaauacugcgguucucaugc.....................   1    1    JCK-1
.........................................................................................................................................................................................................acuaauacugcgguucucaugc.....................   1    0    LT-2
.........................................................................................................................................................................................................acuaauacugcgguucucaugc.....................   5    0    LT-3
.........................................................................................................................................................................................................acuaauacugcgguucucaugc.....................   2    0    LT-1
.........................................................................................................................................................................................................acuaauacugcgguucucaugc.....................   4    0    JCK-2
.........................................................................................................................................................................................................aUuaauacugcgguucucaugc.....................   2    1    JCK-2
.........................................................................................................................................................................................................acuaauacugcgguucucaugc.....................   6    0    JCK-3
.........................................................................................................................................................................................................aUuaauacugcgguucucaugc.....................   2    1    LT-3
.........................................................................................................................................................................................................acuaauacugUgguucucaugc.....................   1    1    LT-3
.........................................................................................................................................................................................................acuaCuacugcgguucucaugc.....................   1    1    LT-3
.........................................................................................................................................................................................................acuaauacugcgguucucaugc.....................   3    0    JCK-1
.........................................................................................................................................................................................................acuaCuacugcgguucucaugc.....................   7    1    LCK-3
.........................................................................................................................................................................................................acuaauacugcgguucucaugca....................   1    0    LT-1
.........................................................................................................................................................................................................acuaauacugcgguucucaugca....................   2    0    LCK-3
..........................................................................................................................................................................................................cuaauacugcgguucuca........................   1    0    LT-2
..........................................................................................................................................................................................................cuaauacugcgguucuca........................   1    0    LT-3
..........................................................................................................................................................................................................cuaauacugcgguucucau.......................   2    0    LT-1
..........................................................................................................................................................................................................cuaauacugcgguucucauU......................   1    1    JCK-1
..........................................................................................................................................................................................................cuaauacugcgguucucauU......................   3    1    JCK-2
..........................................................................................................................................................................................................cuaauacugcgguucucaug......................   4    0    JCK-3
..........................................................................................................................................................................................................cuaauacugcgguucucaug......................   4    0    JCK-1
..........................................................................................................................................................................................................cuaauaUugcgguucucaug......................   1    1    LT-3
..........................................................................................................................................................................................................cuaauacugUgguucucaug......................   1    1    LT-1
..........................................................................................................................................................................................................cuaauacugcgguucucauU......................   2    1    JCK-3
..........................................................................................................................................................................................................cuaauacugcgguucucauU......................   1    1    LCK-3
..........................................................................................................................................................................................................cuaauacugcgguucucaug......................   1    0    JCK-2
..........................................................................................................................................................................................................cuaauacugcUguucucaug......................   2    1    JCK-3
..........................................................................................................................................................................................................Uuaauacugcgguucucaugc.....................   1    1    JCK-2
..........................................................................................................................................................................................................cuaauacugUgguucucaugc.....................   2    1    JCK-2
..........................................................................................................................................................................................................cuaaCacugcgguucucaugc.....................   1    1    LT-3
..........................................................................................................................................................................................................cuaauacugcgguucucaugc.....................   2    0    JT-3
..........................................................................................................................................................................................................Uuaauacugcgguucucaugc.....................   4    1    LT-1
..........................................................................................................................................................................................................cuaauacugcgguucucaugc.....................   25   0    LT-2
..........................................................................................................................................................................................................cuaauacugcggCucucaugc.....................   1    1    LT-1
..........................................................................................................................................................................................................cuaauacugcCguucucaugc.....................   1    1    JCK-2
..........................................................................................................................................................................................................cuaauacugcgguucucaugc.....................   29   0    LCK-3
..........................................................................................................................................................................................................cuCauacugcgguucucaugc.....................   3    1    LT-2
..........................................................................................................................................................................................................Uuaauacugcgguucucaugc.....................   3    1    JCK-3
..........................................................................................................................................................................................................cuaauacugcgguucucaugc.....................   8    0    LT-1
..........................................................................................................................................................................................................Uuaauacugcgguucucaugc.....................   1    1    JCK-1
..........................................................................................................................................................................................................cuaauacugcgguucucaugc.....................   21   0    JCK-1
..........................................................................................................................................................................................................cuaauacugcgguucucGugc.....................   1    1    LT-1
..........................................................................................................................................................................................................cuaCuacugcgguucucaugc.....................   3    1    JCK-3
..........................................................................................................................................................................................................cuaCuacugcgguucucaugc.....................   1    1    LT-3
..........................................................................................................................................................................................................cuaauacugcgguucucaugc.....................   2    0    JT-2
..........................................................................................................................................................................................................cuaauacugUgguucucaugc.....................   1    1    LT-1
..........................................................................................................................................................................................................cuaauacugUgguucucaugc.....................   9    1    LT-2
..........................................................................................................................................................................................................cuCauacugcgguucucaugc.....................   2    1    JCK-1
..........................................................................................................................................................................................................cuaauacugUgguucucaugc.....................   1    1    LT-3
..........................................................................................................................................................................................................cuaauacugcgguucucaugc.....................   41   0    JCK-2
..........................................................................................................................................................................................................cuaauacugcgguucucaugc.....................   13   0    LT-3
..........................................................................................................................................................................................................cuaauacugcgguucucaugc.....................   22   0    JCK-3
..........................................................................................................................................................................................................cuaauacugcgguucucauAca....................   1    1    JCK-1
...........................................................................................................................................................................................................uaauacugcgguucucau.......................   1    0    LT-3
...........................................................................................................................................................................................................uaaCacugcgguucucau.......................   1    1    LT-3
...........................................................................................................................................................................................................uaauacugcgguucucaug......................   2    0    LT-1
...........................................................................................................................................................................................................uaauacugcgguucucaug......................   1    0    LT-3
...........................................................................................................................................................................................................uaauacugUgguucucaugc.....................   1    1    LT-1
...........................................................................................................................................................................................................uaauacugUgguucucaugc.....................   1    1    LT-3
...........................................................................................................................................................................................................uaauacugcgguucucaugc.....................   1    0    LT-3
...........................................................................................................................................................................................................uaauacugcUguucucaugc.....................   1    1    JCK-3
...........................................................................................................................................................................................................uaauacugcgguucucaugc.....................   1    0    LT-1
...........................................................................................................................................................................................................uCauacugcgguucucaugc.....................   6    1    LT-2
...........................................................................................................................................................................................................uaaCacugcgguucucaugc.....................   1    1    JCK-3
...........................................................................................................................................................................................................uaauacugcgguucucaugca....................   1    0    LT-1
...........................................................................................................................................................................................................uaauacugUgguucucaugcag...................   1    1    LT-2
............................................................................................................................................................................................................aauacugcgguucucauU......................   1    1    LT-3
............................................................................................................................................................................................................aauacugcgguucucaug......................   3    0    JT-2
```

```
                                                                                                                        Star                                                                                  Mature
aaauaauaagcucauaucuuguguucaaaguuugaaacuaaaaccccaauuucuuaauuuaucaauauauauauauauauguauauauguauaccguucgucuaugguauggacgguuuucauacggauuacaauauuggugaugauuuuucauaguauuggugacgauuuucuagaaaaucguuguuaauacuauaaaaaaaucaucacuaauacugcgguucucaugcagaccguccucaccauagaau
...................................................................................................................................................................................................................aauacugcgguucucaug......................   5   0   JT-3
...................................................................................................................................................................................................................aauacugcgguucucaugc.....................   2   0   LT-1
...................................................................................................................................................................................................................aaCacugcgguucucaugc.....................   1   1   JCK-2
...................................................................................................................................................................................................................aauacugcgguucuUaugc.....................   1   1   JCK-2
...................................................................................................................................................................................................................aauacugUgguucucaugc.....................   3   1   JCK-3
...................................................................................................................................................................................................................aauacugcgguucucaugc.....................   6   0   JCK-2
...................................................................................................................................................................................................................aauacugUgguucucaugc.....................   1   1   LT-1
...................................................................................................................................................................................................................aauacugcgguucucaugc.....................   6   0   LT-2
...................................................................................................................................................................................................................Uauacugcgguucucaugc.....................   1   1   JCK-2
...................................................................................................................................................................................................................aauacugUgguucucaugc.....................   3   1   JCK-2
...................................................................................................................................................................................................................aauacugUgguucucaugc.....................   1   1   LT-2
...................................................................................................................................................................................................................aauacugUgguucucaugc.....................   5   1   LCK-3
...................................................................................................................................................................................................................aauacugcgguucucaugc.....................   4   0   JCK-3
...................................................................................................................................................................................................................aauacugcgguucucaugc.....................   3   0   JCK-1
...................................................................................................................................................................................................................aauacugcgguucucaugc.....................   7   0   LT-3
...................................................................................................................................................................................................................aauacugUgguucucaugc.....................   1   1   JCK-1
...................................................................................................................................................................................................................aauacugcgguucucauAcaga..................   1   1   LT-1
...................................................................................................................................................................................................................aauacugcgguucucauAcagacc................   1   1   JCK-2
...................................................................................................................................................................................................................aauacugcgguucucauAcagacc................   1   1   JCK-1
...................................................................................................................................................................................................................aauacugcgguucucaugcaAacc................   1   1   LT-3
....................................................................................................................................................................................................................auacugcgguucucaugc.....................   1   0   JCK-2
....................................................................................................................................................................................................................auacugcgguucucaugc.....................   24  0   LCK-2
....................................................................................................................................................................................................................auacugUgguucucaugc.....................   1   1   JCK-1
....................................................................................................................................................................................................................auacugcgguucucaugc.....................   2   0   LT-3
....................................................................................................................................................................................................................auacugcgguucucaugc.....................   22  0   LCK-1
....................................................................................................................................................................................................................auacugcgguucucauAcaga..................   3   1   LT-2
.......................................................................................................................................................................................................................cuAcgguucucaugcaga..................   1   1   LT-2
...........................................................................................................................................................................................................................gguucucaugcagaUcguccuc..........   1   1   LT-2
...........................................................................................................................................................................................................................gguucucaugcagaUcguccuc..........   1   1   LT-3
...........................................................................................................................................................................................................................gAuucucaugcagaccguccuca.........   1   1   LT-3
.............................................................................................................................................................................................................................uucucaugcagaccguccucaUc.......   1   1   JCK-2
..............................................................................................................................................................................................................................ucucaugcagaccguUcuca.........   2   1   LT-3
..............................................................................................................................................................................................................................ucucaugcagacUguccucacc.......   1   1   LT-2
..............................................................................................................................................................................................................................ucucaugcagaccguUcucacca......   1   1   LT-1
...............................................................................................................................................................................................................................cucaugcagacUguccuca.........   1   1   LT-3
...............................................................................................................................................................................................................................cucaugcagacUguccuca.........   4   1   LCK-3
...............................................................................................................................................................................................................................cucaugcagaUcguccuca.........   2   1   JCK-3
...............................................................................................................................................................................................................................cucaugcagacUguccucac........   1   1   LT-1
...............................................................................................................................................................................................................................cucaugcagacUguccucacc.......   2   1   JCK-2
...............................................................................................................................................................................................................................cucauAcagaccguccucacc.......   3   1   LT-3
...............................................................................................................................................................................................................................cucaugcagaUcguccucacc.......   1   1   LT-3
...............................................................................................................................................................................................................................cucaugcagaccguccucaccau.....   1   0   JCK-1
...............................................................................................................................................................................................................................cucaugcagaccguccucaccau.....   1   0   JCK-2
................................................................................................................................................................................................................................ucaugcagaUcguccucacc.......   1   1   LT-3
................................................................................................................................................................................................................................ucaugcagaccguccucacca......   1   0   JCK-1
................................................................................................................................................................................................................................ucaugcagaccguccucacca......   1   0   JCK-2
................................................................................................................................................................................................................................ucaugcagaccguccucacca......   1   0   JCK-3
................................................................................................................................................................................................................................ucaugcagaccguGcucacca......   1   1   LT-3
................................................................................................................................................................................................................................ucaugcagaccgucUucaccau.....   1   1   LT-3
................................................................................................................................................................................................................................ucaugcagaccgucUucaccaua....   1   1   JCK-2
................................................................................................................................................................................................................................ucauAcagaccguccucaccaua....   2   1   LT-3
.................................................................................................................................................................................................................................caugcagaUcguccucac........   1   1   LT-3
.................................................................................................................................................................................................................................caugcagaccguGcucacc.......   1   1   LT-3
.................................................................................................................................................................................................................................caugcagaccgucUucacc.......   3   1   LT-2
.................................................................................................................................................................................................................................caugcagaccguUcucacc.......   1   1   JCK-1
.................................................................................................................................................................................................................................caugcagaccguGcucaccau.....   1   1   LT-3
.................................................................................................................................................................................................................................caugcagacUguccucaccau.....   1   1   LT-1
.................................................................................................................................................................................................................................caugcagaccguccucaccauaga..   1   0   JCK-3
.................................................................................................................................................................................................................................caugcagaUcguccucaccauaga..   2   1   LT-1
.................................................................................................................................................................................................................................cauUcagaccguccucaccauaga..   1   1   JCK-3
..................................................................................................................................................................................................................................augcUgaccguccucacca......   1   1   LT-1
...................................................................................................................................................................................................................................ugcagaccguccucaccauC....   1   1   JCK-1
...................................................................................................................................................................................................................................ugcagacUguccucaccauagaau   1   1   LT-1
....................................................................................................................................................................................................................................gcagaUcguccucaccauagaau   1   1   LT-3
.....................................................................................................................................................................................................................................cagaccgucGucaccauagaau   2   1   LT-2
......................................................................................................................................................................................................................................agaccgucUucaccauaga..   1   1   JCK-2
.........................................................................................................................................................................................................................................ccguccucaccauagaUu   1   1   LT-3
```
